# Head-to-head comparison of Microflex LT and Vitek MS systems for routine identification of microorganisms by MALDI-TOF mass spectrometry in Chile

**DOI:** 10.1371/journal.pone.0177929

**Published:** 2017-05-18

**Authors:** Lorena Porte, Patricia García, Stephanie Braun, María Teresa Ulloa, Mónica Lafourcade, Alisson Montaña, Carolina Miranda, Gerardo Acosta-Jamett, Thomas Weitzel

**Affiliations:** 1 Laboratorio Clínico, Clínica Alemana de Santiago, Facultad de Medicina Clínica Alemana, Universidad del Desarrollo, Santiago, Chile; 2 Unidad de Microbiología, Laboratorio Clínico, Hospital Militar, Santiago, Chile; 3 Departamento de Laboratorios Clínicos, Escuela de Medicina, Pontificia Universidad Católica de Chile, Santiago, Chile; 4 Programa de Microbiología, ICBM Facultad de Medicina, Universidad de Chile, Santiago, Chile; 5 Laboratorio de Microbiología, Clínica Santa María, Santiago, Chile; 6 Laboratorio de Microbiología, Servicio de Laboratorios Clínicos, Red de Salud UC-CHRISTUS, Santiago, Chile; 7 Instituto de Medicina Preventiva Veterinaria, Facultad de Ciencias Veterinarias, Universidad Austral, Valdivia, Chile; Universita degli Studi di Parma, ITALY

## Abstract

**Background:**

Matrix-assisted laser desorption ionization-time of flight (MALDI-TOF) mass spectrometry (MS) is a new and revolutionary identification method for microorganisms and has recently been introduced into clinical microbiology in many industrialized countries in Europe and North America.

**Objectives:**

Our study aimed to compare the performance and practicality of two commercial MALDI-TOF MS platforms in a head-to head manner at a routine laboratory in Chile.

**Methods:**

During a five-month period in 2012–13, the diagnostic efficiency (correct identification rate) and agreement between Microflex LT (Bruker Daltonics) and Vitek MS (bioMérieux) was compared in a parallel manner to conventional identification including genotypic analysis for difficult-to-identify strains. The study included 804 microbial isolates: 252 *Enterobacteriaceae*, 126 non-fermenters, 36 other gram-negative rods, 279 gram-positive cocci, 32 gram-positive rods, 32 anaerobes, and 47 yeasts. Other relevant factors of the two devices such as user friendliness and connectivity were also evaluated and compared.

**Results:**

Both systems correctly identified the vast majority (98%) of the isolates to the genus level. Vitek MS reached higher rates of identification to species and species complex level than Microflex LT (81% vs. 85% and 87% vs. 93%, respectively), which was mainly based on the higher performance among coagulase negative staphylococci and *Candida* isolates. The evaluation of user friendliness and other technical aspects showed only marginal differences, which slightly favored Vitek MS, mainly due to its ready-to-use supplies, easier connectivity and workflow integration, and availability of local technical support.

**Conclusions:**

Both MALDI-TOF MS systems permitted fast and accurate identification of most microbial strains and showed a high level of user-friendliness. The observed differences were marginal and slightly favored Vitek MS, mainly due to practicality and connectivity issues within our setting.

## Introduction

Traditionally, the identification of bacteria and yeasts in clinical samples has relied on phenotypical aspects such as morphology of colonies, microscopical appearance, and biochemical tests, which are time consuming and costly. For species that are difficult to identify by these techniques, the method of choice is genomic analysis including sequencing; still, such methods are not widely available and therefore usually not part of routine identification [1]. Considering these limitations, matrix-assisted laser desorption ionization-time of flight (MALDI-TOF) mass spectrometry (MS) has the potential to revolutionize the identification of microorganisms in routine medical microbiology [2–4]. Its basic principle is the generation of characteristic mass spectra allowing a rapid and reliable identification of clinically relevant microorganisms. The application of this new technique has been subject to several reviews [5–11]. Other recent investigations focused on the identification of certain groups of microorganisms, e.g. nonfermenters [12], anaerobes [13,14], mycobacteria [15], yeasts [16], filamentous fungi [17–20], and difficult-to-identify species [21,22]. Studies also demonstrated a positive clinical impact on antibiotic treatment [23,24] as well as cost-effectiveness [25,26]. Future applications of MALDI-TOF MS such as analysis of antimicrobial resistance [27–30] and epidemiological studies by strain typing [31–34] are increasingly recognized and explored.

At present, two identification systems, Microflex LT (Bruker Daltonics, Bremen, Germany) and Vitek MS (bioMérieux, Marcy l'Etoile, France), are commercially available; both were approved for in vitro diagnostics by the FDA during 2013. Vitek MS was developed and formerly marketed as a prototype named Axima Assurance System (Shimadzu Corporation, Kyoto, Japan). Although a variety of studies have evaluated one of the two devices using different panels of clinical isolates of bacteria and fungi, only few compared the two platforms in a strict manner and under routine conditions. Furthermore, almost all evaluations were performed by laboratories in industrialized countries, mainly in Europe and North America, where skilled technical and IT support is more reliably at hand than in non-industrialized and resource-limited regions.

The goal of the presented study was to directly compare the diagnostic performance and other technical and practical aspects of the Microflex LT and Vitek MS systems under the conditions of a routine laboratory in a non-industrialized country in South America.

## Materials and methods

The study was performed from August 2012 to January 2013 in the clinical laboratory of Clínica Alemana de Santiago, Chile.

### Samples and isolates

A total of 804 strains were included: 252 *Enterobacteriaceae*, 126 nonfermenters, 36 other Gram-negative rods, 279 Gram-positive cocci, 32 Gram-positive rods, 32 anaerobes, and 47 yeasts. Almost all isolates were cultured from routine samples received during a five month period (August 2012 to January 2013) and mostly originated from genitourinary and respiratory tracts and blood culture of ambulatory and hospitalized patients from the participating centers in Santiago, Chile. The panel of tested isolates was complemented by strain collections of the participating laboratories, including 42 reference strains from the American Type Culture Collection or external quality programs (S1 Table).

### Species identification

Strains were cultured on commercial media under conditions that were in accordance with international recommendations [35]. Results of MALDI-TOF identification (ID) were compared to those of conventional methods, which mainly based on macro- and micromorphology and automated biochemical and enzymatic testing on Vitek 2 Compact (bioMérieux). For some isolates, other conventional methods such as API (bioMérieux), other biochemical or enzymatic tests, and serotyping were added. Due to national guidelines, all strains of *Salmonella*, *Shigella*, *Campylobacter* and *Vibrio* were confirmed by the national reference laboratory (Instituto de Salud Pública, Santiago, Chile). Discordant results or isolates with incomplete identification were analyzed using molecular techniques including sequencing.

### MALDI-TOF mass spectrometry analysis

All strains were tested in parallel on the Microflex LT (Bruker Daltonics) and Vitek MS (bioMérieux) devices. Before testing, strains were subcultured on commercial media (bioMérieux) in the laboratory of Clínica Alemana. Facultative anaerobes were grown in trypticase soy agar plus 5% sheep blood under standard conditions. Anaerobic isolates were cultured on Schaedler agar in an anaerobic jar. Yeasts were recovered on Sabouraud agar at 35 ± 2°C in aerobiosis.

After sufficient growth (24 to 48 hours), isolates were identified on Microflex LT and Vitek MS in a strictly parallel manner, i.e. colonies were taken from the same medium at the same time by the same technologist. All runs were done in duplicate following the manufacturer’s instructions.

### Microflex LT (Bruker)

In accordance to the manufacturer´s instructions, a portion of each bacterial colony was smeared onto a 96-well metal target plate. After drying, every spot was covered with 1 μL of matrix solution (α-cyano-4-hydroxycinnamic acid, CHCA). This solution was freshly mixed and stored at room temperature as recommended by the manufacturer. In yeasts, the system required an additional extraction step before applying the matrix solution. One or two colonies were suspended in 300 μL of distilled water, then 900 μL of 70% ethanol was added and, after being briefly vortexed, the Eppendorf microtube was centrifuged for 2 min at 12,000 rpm. The pellet was resuspended in 50 μL of formic acid. Then, 50 μL of acetonitrile was added and, after vortexing vigorously, the tube was centrifuged for 2 min at 12,000 rpm. One μL of the supernatant was applied onto the target plate and allowed to dry at room temperature before adding the CHCA matrix solution. After drying, target plates were placed into the instrument, where they were exposed to a 337-nm nitrogen laser, which generates particle spectra recorded in linear mode within a range of 2 to 20 kDa. These spectra were analyzed by the integrated software, which included MBT 3.0 RTC/OC, MBT Reference Library 3.2.1.1 4010, and Compass 1.3. The software version did not include the security-relevant (SR) database. Each result was accompanied by an ID log score. In accordance with the manufacturer´s recommendations, only scores ≥ 2.0 permitted the species diagnosis, while values between 1.7 and 1.99 could only count as genus identifications (independent of given species); results <1.7 were classified as “no identification”. The Microflex LT algorithm also included a second match species with a corresponding ID score. If these scores differed <10%, we considered the identification as inconclusive as described before [36], and results were counted as identification of genus (if genus of first and second match were equal) or as “no identification” (if genus of first and second match were different). As all tests were done in duplicate, two test results were available. The spot with the highest ID score was counted. Microflex LT instrument was regularly calibrated using the Bruker Daltonics bacterial test standard (BTS) as recommended by the manufacturer.

### VITEK MS (bioMérieux)

All procedures followed the manufacturer’s instructions. The principal methodology equals the Microflex LT device. In short, a fraction of a single colony was smeared with a plastic loop onto a disposable target slide (FlexiMass™, bioMérieux) containing three acquisition groups of 16 spots each, and immediately covered with 1 μL of CHCA matrix solution, provided by the manufacturer [37]. In yeasts, a small portion of a single colony was smeared onto the target plate and covered with 0.5 μL formic acid. After drying at room temperature, CHCA matrix solution was applied [38]. *Escherichia coli* ATCC 8739 strain was used as a calibrator and internal control for each acquisition group. After drying, the target plate was placed into the device and analyzed using the bioMérieux platform Myla™ v2.0. The software calculated confidence values for each tested strain. According to the manufacturer, values between 60.0 and 99.9 indicated a reliable discrimination of species or species group. In contrast to Microflex LT, Vitek MS automatically created average scores for the two spots performed for each strain. To guarantee equal conditions with Microflex LT, we reviewed all isolates with identification scores of less than 99.9 and counted the highest score of the two runs.

### Molecular identification

Strains with conflictive results or inconclusive identification by conventional techniques underwent genotypic analysis, which was performed at the Laboratory of Molecular Microbiology of the Pontificia Universidad Católica, Santiago, Chile. There, DNA was extracted using the QI Amp DNA Mini^®^ Kit (Qiagen, Hilden, Germany). Fungal strains were pretreated with lyticase. For bacterial isolates, PCR was performed using two universal primers for the amplification of a 1380 pb region of the 16S rRNA gene (5´-AGT TTG ATC CTG GCT CAG-3´ [39] and 5´-AGG CCC GGG AAC GTA TTC AC-3´ [40]). For yeasts, a region of the 18S rRNA gene of approximately 600 pb was amplified by primers ITS-1 5'- TCCGTAGGTGAACCTGCGG-3' and ITS-4 5'-TCCTCCGCTTATTGATATGC-3' [41].

Each bacterium PCR mix consisted of 5 μl of the extracted DNA, 1.2 μl of each primer (10 μl), 1.2 μl of dNTPs (10 μM), 5 μl of PCR buffer 10X (Applied Biosystems), 6 μl of MgCl_2_ (25 mM, Applied Biosystems), 0.25 μl de AmpliTaq DNA Polymerase LD (5U/ μl, Applied Biosystems), 3 μl of BSA 5% (New England BioLabs) and water until 50 µl of the final reaction were completed. The fungi PCR mix consisted of 5 μl of the extracted DNA, 1.2 μl of each primer (10 μl), 12.5 μl of premix 2x, 0.21 μl of Go-Taq DNA polymerase (5U/ μl, Promega), and water until 25 μl of the final reaction were completed. For bacteria, amplification was performed in an Applied Biosystems 2720 thermocycler with initial denaturation at 96°C for 3 minutes, followed by 30 cycles of 96°C for 30 seconds, 58°C for 45 seconds and 72°C for 45 seconds, with a final extension of 72°C for 10 minutes; for fungi, initial denaturation at 95°C for 5 minutes, followed by 30 cycles of 96°C for 45 seconds, 56°C for 45 seconds and 72°C for 45 seconds, with a final extension of 72°C for 5 minutes. PCR products were visualized in a 1.5% agarose gel stained with ethidium bromide and purified by Nucleospin® Gel and PCR Clean-up kit (Macherey-Nagel, Düren, Germany). Then, purified DNA was directly sequenced using a Big Dye Terminator v3.1 Cycle Sequencing kit on an ABI Prism 3130 sequencer (both Applied Biosystems, Foster City, CA, USA). The primer used for bacteria was 5’-ATT ACC GCG GCT GCT GG-3’; for fungi, ITS 1. The detected sequences were compared with sequences in the GenBank NCBI (National Center for Biotechnology Information; http://www.ncbi.nlm.nih.gov/genbank) using BLAST software (Blast Internet Services, Pittsboro, NC, USA) and interpreted according to CLSI standards [42].

### Classification of results

MALDI-TOF results were compared to those of conventional identification and, in difficult-to-identify strains, to those of molecular methods and included the microbial species (or species complex) and genus; subspecies identifications were not incorporated. To stratify the comparison, MALDI-TOF results were classified into six categories: “correct identification of species”, “correct identification of species complex”, “correct identification of genus”, “no identification”, “misidentification of species”, and “misidentification of genus”. If conventional and molecular methods were unable to identify a strain to the species level, MALDI-TOF results were classified accordingly: for strains that even by molecular methods were only identifiable to the species complex level, the highest category was “correct identification of species complex”, for strains that were only identifiable to the genus level, the best result was “correct identification of genus”. If a species was not included in the database of the respective MS device, a wrongly given species was classified as “correct identification of genus” and not as “misidentification of species”. If the respective device’s instructions clearly indicated a limitation of its capacity to identify a species, species complex or genus, misidentifications were interpreted as “correct identification of species complex” (if species was wrong), “correct identification of genus” (if species and species complex were wrong) or “no identification” (if genus was wrong).

### Comparison of practicality and other factors

Three staff members independently rated their working experience with both instruments using a questionnaire covering the following aspects: user friendliness (including ergonomic design), ready-to-use components (matrix solution and disposable slides), ease of spotting colony onto targets, capacity of target analysis, ease of software use, and workflow integration; other factors referred to the time to get results (including preparation of work list, vacuum time, identification time, and additional extraction steps for yeasts), quality control, availability of technical support, and costs for device, reactants and maintenance.

### Statistical analysis

Statistical analysis was carried out using a DAG Stat spread sheet [43]. The diagnostic efficiency (or correct identification rate) was determined for both systems and compared using McNemar’s test. In order to reach higher numbers of isolates, only groups of microorganisms were analyzed. Overall percent agreement (concordance) between both diagnostic devices was calculated by dividing the number of isolates with equal results by the total number of isolates and by using Kappa tests [44].

### Ethics statement

**Fig 1:**
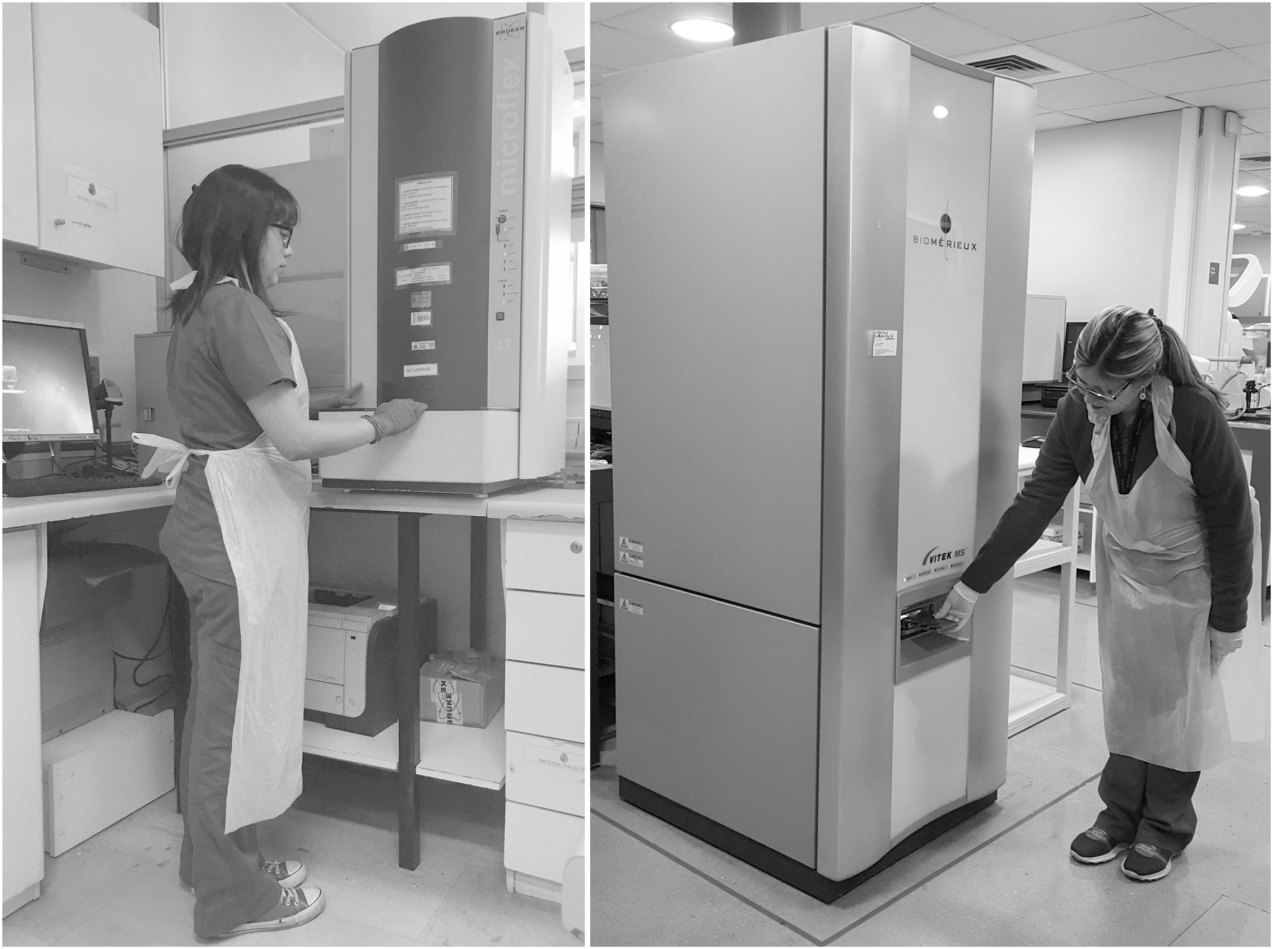
Size and loading position of Microflex LT (left) and Vitek MS (right).

The study was approved by the Institutional Review Board of Universidad del Desarrollo, Santiago, Chile. Since microbial isolates derived from routine diagnostic procedures, informed consents were not obtained. The individuals shown in Fig 1 have given written informed consent (as outlined in PLOS consent form) to publish these photos.

## Results

Of the 804 isolates, Microflex LT and Vitek MS correctly identified 98% strains to the genus level, while the correct species and species complex level were reached in 81% vs. 85% and 87% vs. 93%, respectively. Microflex LT and Vitek MS were unable to identify 1.7% (n = 14) of strains and misidentified 0.5% (n = 4) and 0.6% (n = 5) of isolates, respectively. The comparison of these results revealed that the higher diagnostic efficiency of Vitek MS was mainly due to better performance among Gram-positive cocci (Table 1). Despite these differences, the overall concordance between both systems was 89.5% with a Kappa index of 0.65, indicating substantial strength of agreement (Table 2).

**Table 1:** Diagnostic efficiency of Microflex LT and Vitek MS to identify microbial isolates.

		Microflex LT			Vitek MS			McNemar´s Test	
	Correct identification	n	Efficiency	CI 95%	n	Efficiency	CI 95%	x2	p
**All** (n = 804)	to species level	650	81%	78–84	687	85%	83–88	18.8	<0.0001
	to species complex level	696	87%	84–89	749	93%	91–95	35.1	<0.0001
	to genus level	786	98%	96–99	785	98%	96–99	0.00	1.00
**Gram-negative aerobic bacteria** (n = 414)	to species level	316	76%	72–80	320	77%	73–81	0.4	0.56
	to species complex level	362	87%	84–90	373	90%	87–93	3.5	0.06
	to genus level	402	97%	95–98	401	97%	95–98	1.3	0.25
**Gram-positive cocci** (n = 279)	to species level	245	88%	83–91	269	96%	94–98	20.4	<0.0001
	to species complex level	245	88%	83–91	276	99%	97–100	27.3	<0.0001
	to genus level	278	99%	98–100	278	99%	98–100	0.48	0.50
**Other microorganisms** (n = 111)	to species level	89	80%	72–87	98	88%	81–94	3.8	0.052
	to species complex level	89	80%	72–87	100	90%	83–95	6.7	<0.01
	to genus level	106	96%	90–99	106	96%	90–99	0.25	0.62

CI, confidence interval

**Table 2:** Diagnostic agreement (concordance) of Microflex LT and Vitek MS.

	Microflex LT					
Vitek MS	Species ID +	Complex ID +	Genus ID +	No ID	Species ID -	Genus ID -
Species ID +	634	0	47	3	2	1
Complex ID +	4	46	12	0	0	0
Genus ID +	8	0	28	0	0	0
No ID	1	0	2	11	0	0
Species ID -	3	0	1	0	1	0
Genus ID -	0	0	0	0	0	0

ID +, correct identification; ID -, misidentification

Overall percent agreement: (634+46+28+11+1)/804*100 = 89.5%

Kappa test = 0.65 (0.58–0.72)

The 804 microbial isolates of our study included 139 species, which were represented by one to 96 strains (Table 3). At total of 43 strains had discordant ID results and were further identified by genotypic methods (Table 4). The complete dataset of results is available as Supplemental Information (S1 Table).

**Table 3:** Microflex LT and Vitek MS identification results of 804 isolates compared to reference identification.

* *		Microflex LT						Vitek MS					
Species	N	Species ID +	Complex ID +	Genus ID +	No ID	Species ID -	Genus ID -	Species ID +	Complex ID +	Genus ID +	No ID	Species ID -	Genus ID -
***Enterobacteriaceae***	**252**	**200**	**17**	**24**	**10**	**0**	**1**	**199**	**18**	**23**	**9**	**3**	**0**
*Citrobacter braakii*	2	2	0	0	0	0	0	2	0	0	0	0	0
*Citrobacter farmeri*	1	1	0	0	0	0	0	1	0	0	0	0	0
*Citrobacter freundii*	11	11	0	0	0	0	0	7	1	0	0	3	0
*Citrobacter koseri*	4	4	0	0	0	0	0	4	0	0	0	0	0
*Citrobacter youngae*	1	1	0	0	0	0	0	1	0	0	0	0	0
*Enterobacter aerogenes*	1	1	0	0	0	0	0	1	0	0	0	0	0
*Enterobacter cloacae complex*	17	0	17	0	0	0	0	0	17	0	0	0	0
*Escherichia coli*	96	95	0	1	0	0	0	96	0	0	0	0	0
*Hafnia alvei*	1	1	0	0	0	0	0	1	0	0	0	0	0
*Klebsiella oxytoca*	12	11	0	0	1	0	0	12	0	0	0	0	0
*Klebsiella pneumoniae*	33	33	0	0	0	0	0	33	0	0	0	0	0
*Morganella morganii*	5	5	0	0	0	0	0	5	0	0	0	0	0
*Pantoea agglomerans*	1	0	0	1	0	0	0	1	0	0	0	0	0
*Plesiomonas shigelloides*	1	1	0	0	0	0	0	1	0	0	0	0	0
*Proteus mirabilis*	15	15	0	0	0	0	0	15	0	0	0	0	0
*Proteus vulgaris*	4	4	0	0	0	0	0	0	0	4	0	0	0
*Providencia alcalifaciens*	1	1	0	0	0	0	0	1	0	0	0	0	0
*Providencia rettgeri*	1	1	0	0	0	0	0	1	0	0	0	0	0
*Providencia stuartti*	1	1	0	0	0	0	0	1	0	0	0	0	0
*Raoultella ornithinolytica/planticola*	1	0	0	1	0	0	0	0	0	1	0	0	0
*Raoutella ornithinolytica*	1	0	0	0	0	0	1	1	0	0	0	0	0
*Salmonella enterica (non-typhoidal)*	16	0	0	16	0	0	0	0	0	16	0	0	0
*Salmonella enterica (typhoidal)*	5	0	0	5	0	0	0	3	0	2	0	0	0
*Serratia liquefaciens*	1	1	0	0	0	0	0	1	0	0	0	0	0
*Serratia marcescens*	8	8	0	0	0	0	0	8	0	0	0	0	0
*Shigella sonnei*	9	0	0	0	9	0	0	0	0	0	9	0	0
*Yersinia enterocolitica*	3	3	0	0	0	0	0	3	0	0	0	0	0
**Nonfermenters**	**126**	**88**	**29**	**8**	**0**	**1**	**0**	**90**	**35**	**0**	**1**	**0**	**0**
*Achrobacter xylosoxidans*	1	1	0	0	0	0	0	0	1	0	0	0	0
*Acinetobacter baumannii*	27	0	22	5	0	0	0	0	27	0	0	0	0
*Acinetobacter calcoaceticus*	3	0	3	0	0	0	0	0	3	0	0	0	0
*Acinetobacter haemolyticus*	1	1	0	0	0	0	0	1	0	0	0	0	0
*Acinetobacter junii*	1	1	0	0	0	0	0	1	0	0	0	0	0
*Acinetobacter lwoffii*	1	1	0	0	0	0	0	1	0	0	0	0	0
*Bordetella pertussis*	1	0	0	1	0	0	0	1	0	0	0	0	0
*Burkholderia cepacia/cenocepacia*	4	0	4	0	0	0	0	0	4	0	0	0	0
*Burkholderia multivorans*	1	1	0	0	0	0	0	1	0	0	0	0	0
*Chryseobacterium indologenes*	1	1	0	0	0	0	0	1	0	0	0	0	0
*Elizabethkingia meningoseptica*	2	2	0	0	0	0	0	2	0	0	0	0	0
*Massilia timonae*	1	1	0	0	0	0	0	0	0	0	1	0	0
*Moraxella catarrhalis*	7	7	0	0	0	0	0	7	0	0	0	0	0
*Ochrobactrum anthropi*	2	2	0	0	0	0	0	2	0	0	0	0	0
*Oligella urethralis*	2	2	0	0	0	0	0	2	0	0	0	0	0
*Pseudomona aeruginosa*	52	52	0	0	0	0	0	52	0	0	0	0	0
*Pseudomonas putida*	2	1	0	0	0	1	0	2	0	0	0	0	0
*Stenotrophomonas maltophilia*	17	15	0	2	0	0	0	17	0	0	0	0	0
**Other Gram-negative rods**	**36**	**28**	**0**	**8**	**0**	**0**	**0**	**31**	**0**	**5**	**0**	**0**	**0**
*Aeromonas hydrophila*	2	0	0	2	0	0	0	0	0	2	0	0	0
*Aeromonas media*	1	1	0	0	0	0	0	0	0	1	0	0	0
*Aeromonas spp*.	1	0	0	1	0	0	0	0	0	1	0	0	0
*Aeromonas veronii complex*	1	0	0	1	0	0	0	0	0	1	0	0	0
*Aggregatibacter aphrophilus*	2	1	0	1	0	0	0	2	0	0	0	0	0
*Campylobacter jejuni*	3	3	0	0	0	0	0	3	0	0	0	0	0
*Eikenella corrodens*	2	2	0	0	0	0	0	2	0	0	0	0	0
*Haemophilus influenzae*	10	10	0	0	0	0	0	10	0	0	0	0	0
*Haemophilus parainfluenzae*	2	1	0	1	0	0	0	2	0	0	0	0	0
*Kingella kingae*	2	2	0	0	0	0	0	2	0	0	0	0	0
*Neisseria elongata*	1	0	0	1	0	0	0	1	0	0	0	0	0
*Neisseria gonorrhoeae*	2	2	0	0	0	0	0	2	0	0	0	0	0
*Neisseria meningitidis*	2	2	0	0	0	0	0	2	0	0	0	0	0
*Pasteurella multocida*	1	1	0	0	0	0	0	1	0	0	0	0	0
*Vibrio cholerae*	1	0	0	1	0	0	0	1	0	0	0	0	0
*Vibrio fluvialis*	1	1	0	0	0	0	0	1	0	0	0	0	0
*Vibrio parahaemolyticus*	2	2	0	0	0	0	0	2	0	0	0	0	0
**Gram-positive cocci**	**279**	**245**	**0**	**33**	**0**	**1**	**0**	**269**	**7**	**2**	**0**	**1**	**0**
*Enterococcus avium*	1	0	0	1	0	0	0	1	0	0	0	0	0
*Enterococcus casseliflavus*	3	2	0	1	0	0	0	3	0	0	0	0	0
*Enterococcus faecalis*	38	37	0	1	0	0	0	38	0	0	0	0	0
*Enterococcus faecium*	21	21	0	0	0	0	0	21	0	0	0	0	0
*Enterococcus gallinarum*	3	2	0	0	0	1	0	3	0	0	0	0	0
*Enterococcus gilvus*	1	1	0	0	0	0	0	0	0	1	0	0	0
*Enterococcus hirae*	2	2	0	0	0	0	0	2	0	0	0	0	0
*Enterococcus raffinosus*	1	1	0	0	0	0	0	1	0	0	0	0	0
*Kocuria kristinae*	1	0	0	1	0	0	0	1	0	0	0	0	0
*Lactococcus lactis*	1	1	0	0	0	0	0	1	0	0	0	0	0
*Staphylococcus aureus*	54	54	0	0	0	0	0	54	0	0	0	0	0
*Staphylococcus capitis*	4	4	0	0	0	0	0	4	0	0	0	0	0
*Staphylococcus caprae*	1	0	0	1	0	0	0	1	0	0	0	0	0
*Staphylococcus epidermidis*	43	36	0	7	0	0	0	43	0	0	0	0	0
*Staphylococcus haemolyticus*	3	1	0	2	0	0	0	2	0	0	0	1	0
*Staphylococcus hominis*	8	6	0	2	0	0	0	8	0	0	0	0	0
*Staphylococcus lugdunensis*	5	4	0	1	0	0	0	5	0	0	0	0	0
*Staphylococcus pasteuri/warneri*	1	0	0	1	0	0	0	0	0	1	0	0	0
*Staphylococcus saprophyticus*	5	2	0	3	0	0	0	5	0	0	0	0	0
*Staphylococcus warneri*	4	4	0	0	0	0	0	4	0	0	0	0	0
*Streptococcus agalactiae*	16	15	0	1	0	0	0	16	0	0	0	0	0
*Streptococcus anginosus*	5	5	0	0	0	0	0	5	0	0	0	0	0
*Streptococcus canis*	1	1	0	0	0	0	0	1	0	0	0	0	0
*Streptococcus constellatus*	4	3	0	1	0	0	0	4	0	0	0	0	0
*Streptococcus dysgalactiae*	4	4	0	0	0	0	0	4	0	0	0	0	0
*Streptococcus gallolyticus*	5	5	0	0	0	0	0	5	0	0	0	0	0
*Streptococcus lutetiensis*	1	1	0	0	0	0	0	1	0	0	0	0	0
*Streptococcus mitis*	1	0	0	1	0	0	0	0	1	0	0	0	0
*Streptococcus mitis/oralis*	6	0	0	6	0	0	0	0	6	0	0	0	0
*Streptococcus parasanguinis*	2	1	0	1	0	0	0	2	0	0	0	0	0
*Streptococcus pneumoniae*	16	15	0	1	0	0	0	16	0	0	0	0	0
*Streptococcus pyogenes*	13	13	0	0	0	0	0	13	0	0	0	0	0
*Streptococcus salivarius*	1	1	0	0	0	0	0	1	0	0	0	0	0
*Streptococcus sanguinis*	3	3	0	0	0	0	0	3	0	0	0	0	0
*Streptococcus thermophilus*	1	0	0	1	0	0	0	1	0	0	0	0	0
**Gram-positive rods**	**32**	**23**	**0**	**7**	**1**	**1**	**0**	**20**	**2**	**6**	**3**	**1**	**0**
*Actinomyces* spp.	3	0	0	3	0	0	0	0	0	3	0	0	0
*Bacillus cereus*	1	1	0	0	0	0	0	0	1	0	0	0	0
*Corynebacterium coyleae*	1	1	0	0	0	0	0	1	0	0	0	0	0
*Corynebacterium kroppenstedtii*	1	0	0	1	0	0	0	0	0	0	1	0	0
*Corynebacterium* spp.	1	0	0	1	0	0	0	0	0	0	1	0	0
*Corynebacterium striatum*	6	6	0	0	0	0	0	6	0	0	0	0	0
*Corynebacterium striatum/simulans*	1	0	0	0	0	1	0	0	0	0	0	1	0
*Corynebacterium tuberculostearicum*	1	1	0	0	0	0	0	1	0	0	0	0	0
*Erysipelotrix rhusiopathiae*	1	1	0	0	0	0	0	1	0	0	0	0	0
*Lactobacillus gasseri*	1	1	0	0	0	0	0	0	1	0	0	0	0
*Leifsonia aquatica*	1	0	0	1	0	0	0	1	0	0	0	0	0
*Listeria monocytogenes*	9	9	0	0	0	0	0	9	0	0	0	0	0
*Microbacterium* spp.	1	0	0	1	0	0	0	0	0	1	0	0	0
*Nocardia neocaledoniensis*	1	0	0	0	1	0	0	0	0	0	1	0	0
*Paenibacillus macerans*	1	1	0	0	0	0	0	0	0	1	0	0	0
*Paenibacillus polymyxa*	1	1	0	0	0	0	0	0	0	1	0	0	0
*Trueperella bernardiae*	1	1	0	0	0	0	0	1	0	0	0	0	0
**Anaerobes**	**32**	**29**	**0**	**3**	**0**	**0**	**0**	**32**	**0**	**0**	**0**	**0**	**0**
*Bacteroides fragilis*	4	4	0	0	0	0	0	4	0	0	0	0	0
*Bacteroides ovatus*	2	2	0	0	0	0	0	2	0	0	0	0	0
*Bacteroides thetaiotaomicron*	3	3	0	0	0	0	0	3	0	0	0	0	0
*Bacteroides vulgatus*	1	1	0	0	0	0	0	1	0	0	0	0	0
*Clostridium difficile*	2	2	0	0	0	0	0	2	0	0	0	0	0
*Clostridium perfringens*	5	5	0	0	0	0	0	5	0	0	0	0	0
*Clostridium septicum*	1	1	0	0	0	0	0	1	0	0	0	0	0
*Clostridium sordellii*	1	1	0	0	0	0	0	1	0	0	0	0	0
*Clostridium tertium*	1	1	0	0	0	0	0	1	0	0	0	0	0
*Fusobacterium necrophorum*	2	1	0	1	0	0	0	2	0	0	0	0	0
*Fusobacterium nucleatum*	1	0	0	1	0	0	0	1	0	0	0	0	0
*Prevotella bivia*	2	2	0	0	0	0	0	2	0	0	0	0	0
*Prevotella melaninogenica*	1	1	0	0	0	0	0	1	0	0	0	0	0
*Propionibacterium acnes*	4	3	0	1	0	0	0	4	0	0	0	0	0
*Veillonella parvula*	2	2	0	0	0	0	0	2	0	0	0	0	0
**Yeasts**	**47**	**37**	**0**	**7**	**3**	**0**	**0**	**46**	**0**	**0**	**1**	**0**	**0**
*Candida albicans*	24	19	0	5	0	0	0	24	0	0	0	0	0
*Candida dubliniensis*	1	1	0	0	0	0	0	1	0	0	0	0	0
*Candida glabrata*	3	3	0	0	0	0	0	3	0	0	0	0	0
*Candida guillermondii*	1	1	0	0	0	0	0	1	0	0	0	0	0
*Candida krusei*	3	3	0	0	0	0	0	3	0	0	0	0	0
*Candida lusitaniae*	2	1	0	0	1	0	0	1	0	0	1	0	0
*Candida parapsilosis*	2	2	0	0	0	0	0	2	0	0	0	0	0
*Candida tropicalis*	8	5	0	2	1	0	0	8	0	0	0	0	0
*Cryptococcus neoformans*	2	1	0	0	1	0	0	2	0	0	0	0	0
*Saccharomyces cereviciae*	1	1	0	0	0	0	0	1	0	0	0	0	0
**TOTAL**	**804**	**650**	**46**	**90**	**14**	**3**	**1**	**687**	**62**	**36**	**14**	**5**	**0**

**Table 4:** Identification results of discordant strains.

Conventional identification	MALDI-TOF MS identification				Genotypic identification
Vitek 2	Microflex LT	C	Vitek MS	C	16S rRNA sequencing
*Acinetobacter baumannii*	*A*. genomspecies 3	2	*A*. *baumannii* complex	2	*A*. *calcoaceticus*
*Acinetobacter baumannii*	*A*. genomspecies 3	2	*A*. *baumannii* complex	2	*A*. *calcoaceticus*
*Acinetobacter baumannii*	*A*. genomspecies 3	2	*A*. *baumannii* complex	2	*A*. *calcoaceticus*
*Actinomyces meyeri*	*A*. *odontolyticus*	3	*A*. *odontolyticus*	3	*Actinomyces* sp.
*Actinomyces naeslundi*	*Actinomyces* spp.	3	*Actinomyces viscosus*	3	*Actinomyces* sp.
*Aeromonas hydrophila*	*A*. *caviae*	3	*A*. *hydrophila/caviae*	3	*A*. *hydrophila*
*Aeromonas hydrophila*	*A*. *caviae*	3	*A*. *hydrophila/caviae*	3	*A*. *hydrophila*
*Aeromonas hydrophila*	*A*. *caviae/hydrophila*	3	*A*. *hydrophila/caviae*	3	*Aeromonas* sp.
*Aeromonas salmonicida*	*Paenibacillus macerans*	1	*Paenibacillus* spp.	3	*P*. *macerans*
*Aeromonas sobria*	*A*. *hydrophila/caviae*	3	*A*. *hydrophila/caviae*	3	*A*. *veronii* complex
*Aeromonas* spp.	*A*. *media*	1	*A*. *hydrophila/caviae*	3	*A*. *media*
*Arcanobacterium bernardiae*	*Trueperella bernardiae*	1	*T*. *bernardiae*	1	*T*. *bernardiae*
*Bacillus* sp	*Microbacterium maritypicum/liquefaciens*	3	*M*. *oxydans*	3	*Microbacterium* sp.
*Bordetella pertussis*	*B*. *bronchiseptica*	3	*B*. *pertussis*	1	*B*. *pertussis*
*Brevundimona diminuta*	*Massilia timonae*	1	no identification	4	*M*. *timonae*
*Burkholderia cepacia*	*B*. *cenocepacia*	2	*B*. *cepacia*	2	*B*. *cepacia/cenocepacia*
*Burkholderia cepacia*	*B*. *cenocepacia/vietnamiensis*	2	*B*. *cepacia*	2	*B*. *cepacia/cenocepacia*
*Burkholderia cepacia*	*B*. *cenocepacia*	2	*B*. *cepacia*	2	*B*. *cepacia/cenocepacia*
*Candida dubliniensis/parapsilosis*	no identification	4	no identification	4	*C*. *lusitaniae*
*Citrobacter freundii*	*C*. *freundii*	1	*C*. *werkmanii*	5	*C*. *freundii*
*Citrobacter freundii*	*C*. *freundii*	1	*C*. *braakii*	5	*C*. *freundii*
*Citrobacter youngae*	*C*. *freundii*	1	*C*. *braakii*	5	*C*. *freundii*
*Clostridium sordellii*	*Bacteroides ovatus*	1	*B*. *ovatus*	1	*B*. *ovatus*
*Corynebacterium amycolatum/diphtheriae*	*C*. *accolens*	3	no identification	4	*Corynebacterium* sp.
*Corynebacterium minutissimum*	*C*. *kroppenstedtii*	3	no identification	4	*C*. *kroppenstedtii*
*Corynebacterium pseudodiphtheriticum*	*C*. *propinquum*	5	*C*. *propinquum*	5	*C*. *striatum/simulans*
*Enterobacter cloacae*	*E*. *asburiae*	2	*E*.*cloacae/asburiae*	2	*E*.*cloacae*
*Enterobacter cloacae*	*E*. *kobei/asburiae*	2	*E*.*cloacae/asburiae*	2	*E*.*cloacae*
*Enterobacter cloacae*	*E*. *asburiae*	2	*E*.*cloacae/asburiae*	2	*Enterobacter* spp.
*Enterobacter hormaechei*	*E*. *cloacae*	2	*E*.*cloacae/asburiae*	2	*E*. *hormaechei*
*Enterococcus casseliflavus*	*E*. *gilvus/devriesei*	1	*E*.*gallinarum*	3	*E*. *gilvus*
*Enterococcus faecium*	*E*. *faecium*	5	*E*. *gallinarum*	1	*E*. *gallinarum*
*Klebsiella oxytoca*	*K*. *oxytoca/R*. *ornithinolytica*	4	*K*. *oxytoca*	1	*K*. *oxytoca*
*Klebsiella pneumoniae*	*K*. *pneumoniae*	6	*Raoutella ornithinolytica*	1	*R*. *ornithinolytica*
*Leifsonia/Chromobacterium*	*L*. *aquatica*	3	*L*. *aquatica*	1	*L*. *aquatica*
*Nocardia* spp.	no identification	4	no identification	4	*N*. *neocaledoniensis*
*Prevotella oralis*	*Bacteroides thetaiotaomicron*	1	*B*. *thetaiotaomicron*	1	*B*. *thetaiotaomicron*
*Pseudomonas putida*	*P*. *monteilii*	5	*P*. *putida*	1	*P*. *putida*
*Raoultella planticola/ornithinolytica*	*R*. *ornithinolytica/planticola*	3	*R*. *planticola*	3	*R*. *ornithinolytica/planticola*
*Staphylococcus warneri*	*S*. *warneri*	3	*S*. *warneri*	5	*S*. *haemolyticus*
*Staphylococcus warneri*	*S*. *pasteuri/warneri*	3	*S*. *pasteuri*	3	*S*. *pasteuri/warneri*
*Streptococcus mitis*	*S*. *pneumoniae*	3	*S*. *mitis/oralis*	2	*S*. *mitis/oralis*
*Streptococcus viridans*	*S*. *pneumoniae*	3	*S*. *mitis/oralis*	2	*S*. *mitis*

C, classification of MALDI-TOF ID results as 1 (correct species), 2 (correct species complex), 3 (correct genus), 4 (no ID), 5 (misidentification species), and 6 (misidentification genus)

### Enterobacteriaceae

Both systems correctly identified 86% (217/252) of strains to the species or species complex level (Table 3). Most identifications as “species complex” occurred within the *Enterobacter cloacae* complex. Since, according to the manufacturers, both MALDI-TOF systems are incapable of distinguishing among the six species of this complex, results were not further analyzed and classified as “correct identification of species complex”. Another known limitation of both devices affected the discrimination of *Salmonella* serovars. Although both manufacturers recommend additional serovar testing and mostly report the isolates as *Salmonella* sp., Vitek MS was able to correctly identify three of the five typhoidal serovars, which were counted as correct identification of species (Table 3, S1 Table). Another problematic enteropathogen for MALDI-TOF identification was *Shigella*. All nine strains of *Shigella sonnei* were misidentified as *E*. *coli* by both devices. Since results were accompanied by a comment indicating the inability to discriminate *Shigella* from *E*. *coli*, results were classified as “without identification” as in previous evaluations [37]. Other problems occurred with *Proteus vulgaris* isolates, which were all identified as *P*. *vulgaris/penneri* with a 50/50 score by Vitek MS, and with *Citrobacter freundii*, which was misidentified (as different *Citrobacter* species) by Vitek MS in three of 11 strains (Table 4, S1 Table).

### Nonfermenters

The vast majority of nonfermenters were correctly identified by both systems to the species or species complex levels (Table 3). Species complex ID was mainly reported among *Acinetobacter baumannii* complex and *Burkholderia cepacia*/*cenocepacia* strains. Biochemical and molecular reference methods of our study could also not differentiate these species. Therefore, all MALDI-TOF results were classified as “correct identification of species complex” (Table 4, S1 Table).

Since according to its instructions, Microflex LT was incapable to distinguish between *Bordetella* species, misidentification of *B*. *pertussis* as *B*. *bronchioseptica* was counted as “correct identification of genus”. Two of the 17 *Stenotrophomonas maltophilia* isolates were classified as “correct identification of genus” by Microflex LT, because of insufficient ID scores (<2.0). Misidentifications occurred with a *Massilia timonae* isolate, which was unidentifiable by Vitek MS, and with a *Pseudomonas putida* strain, which was misidentified by Microflex LT as *P*. *monteilii* (Table 4)

### Other Gram-negative rods

Within this heterogeneous group, all isolates were correctly identified to the genus level by both systems (Table 3). Difficulties to determine the species affected all except one *Aeromonas* isolate, which was diagnosed by Microflex LT as *A*. *media*. Notably, also our sequencing methods reached a conclusive species results in only two of five *Aeromonas* strains (Table 4). In the case of *Vibrio* spp., both systems performed well. The only isolate of *Vibrio cholerae* was identified by Microflex LT as *V*. *albensis*, but as *V*. *cholerae* was not included in the available database, the result was counted as “correct identification of genus”.

### Gram-positive cocci

This group consisted of 279 isolates of staphylococci, enterococci, and streptococci, of which 245 (88%) and 276 (99%) were correctly identified to the species/species complex levels by Microflex LT and Vitek MS, respectively (Table 3). Still, the diagnostic efficiency of Microflex LT for species or species complex was inferior to Vitek MS (Table 1).

Both systems reached high rates of correct species IDs in the most common enterococcal species, *E*. *faecalis* and *E*. *faecium*; only one of 59 strains was not identified as the correct species by Microflex LT. This system was also less capable of identifying the uncommon species, with two genus level identifications and one species misidentification (Table 3).

Vitek MS had a very good performance among staphylococci, except for one strain of *S*. *haemolyticus* misidentified as *S*. *warneri* (Table 4). Microflex LT, on the other hand, performed well with *S*. *aureus*, but only reached genus identification level in various coagulase negative staphylococci (7/43 *S*. *epidermidis*, 1/1 *S*. *caprae*, 2/8 *S*. *hominis*, 1/1 *S*. *lugdunensis*, and 3/5 *S*. *saprophyticus*).

All streptococcal isolates were identified to species or species complex level by Vitek MS. Microflex LT was less accurate especially among viridans streptococci; most relevantly, six of seven strains of *S*. *mitis* and *S*. *mitis/oralis*, were reported as *S*. *pneumoniae* (S1 Table). Since, according to the Microflex LT manual, a confirmatory test has to be used for *S*. *pneumoniae*, these results were counted as “correct genus identification”.

### Gram-positive rods

This heterogeneous group of microorganisms includes various genera and species, which are difficult-to-identify by traditional phenotypical methods [45]. Of the 32 strains of our study, 23 (72%) and 22/32 (69%) were correctly identified to the species or species complex levels by Microflex LT and Vitek MS, respectively; further seven (22%) and six (19%) isolates were classified as “correct genus identification”, respectively (Table 3). All *L*. *monocytogenes* isolates were diagnosed by both devices to the species level. The species of the included *Actinomyces* strains were not identifiable by our reference methods; MALDI-TOF results were therefore only counted as “correct genus” (Table 4, S1 Table). The species *N*. *neocaledoniensis* was not included within the databases of either system, resulting in “no identification”.

### Anaerobes

Both systems displayed an excellent performance for the identification of the 32 isolates of anaerobes (Table 3), of which seven (22%) represented reference stains (S1 Table). All isolates were correctly identified to the species level, except for three strains, which were reported to the genus level by Microflex LT.

### Yeasts

Of the 47 included isolates, 51% were *C*. *albicans* (Table 3). The diagnostic performance of Vitek MS was very high; with the exception of one isolate (*C*. *lusitaniae*), which was not identifiable, all species were correctly recognized. Microflex LT, on the other hand, diagnosed seven isolates only to the genus level and did not provide any identification in three additional isolates (*C*. *tropicalis*, *C*. *lusitaniae*, *and Cryptococcus neoformans*).

### Comparison of practicality

The comparison of both devices regarding their user friendliness, speed, costs, and other aspects is summarized in Table 5.

**Table 5:** Comparison of practicality and other technical aspects of Microflex LT and Vitek MS.

	Microflex LT	Vitek MS
**User friendliness**		
Dimensions of device	**☺☺☺**	**☺**
Ergonomic design	**☺☺**	**☺**
Noise emission	Low	High
Ease of smear preparation	**☺☺**	**☺**
Disposable target	No	Yes
Ready-to use matrix solution	No	Yes
Sample capacity per run	16	4x48
Workflow integration*	**☺**	**☺☺**
**Identification time**		
Prepare work list	2 min	2 min
Create vacuum	3 min	6 min
Identification (n° of sample)	50 min (96)	45 min (48)
Results in real-time manner	Per spot	Per 16 spots
Yeasts extraction step	20 min	0
**Costs**		
Device	**☺**	**☺**
Disposables/reactants	**☺☺☺**	**☺☺**
Maintenance	**☺☺**	**☺**
**Other aspects**		
Calibration	Every 96 spots	Every 16 spots
Traceability	**☺**	**☺☺**
Local technical support*	**☺**	**☺☺**
Remote technical support	**☺☺**	**☺☺**

**☺**, acceptable; **☺☺**, good; **☺☺☺**, very good

*Considering situation in Chile

### User friendliness

Microflex LT as a benchtop instrument required less laboratory space and was more comfortable to work with (e.g. loading of targets). Vitek MS, on the other hand, is a floor-mounted appliance that occupies larger space and, since the loading station is in a low position, personnel have to bend down to introduce the targets (Fig 1). Smear preparation with Microflex LT was less meticulous and required less experience compared to Vitek MS. The availability of disposable targets and ready-to-use matrix solution of the Vitek MS system was an advantage, since it reduced pre-analytical steps and possible errors. Vitek MS permits simultaneous loading of four targets, which allows preparation of up to 192 spots in four different work stations in parallel. Vitek MS was easier to integrate into the workflow, using a common middleware (Myla™, bioMérieux) with other routine devices of our lab (Vitek Compact, bioMérieux). Microflex LT, on the other hand, required the development of a novel interphase with the local Laboratory Information System.

#### Speed

Microflex LT was faster creating the vacuum and identifying the samples. It also permitted to access real-time results. On the other hand, Vitek MS did not cause delays due to additional extraction steps before yeast identification.

#### Costs

Both systems had similarly high acquisition costs. However, Microflex LT reactants and maintenance were less expensive than for Vitek MS.

#### Other aspects

The quality control protocol of both devices included automated calibration steps using reference strains, which had a higher frequency in Vitek MS. This system also permitted electronic traceability of results, which, at the time of the study, was not possible with Microflex LT. Another aspect within the implementation of these new diagnostic devices was the availability of local technical support. While bioMérieux (Vitek MS) maintained a local office in Chile, Bruker (Microflex LT), at the time of the study, had its nearest technical support facility in the USA.

## Discussion

With the introduction of MALDI-TOF MS, clinical diagnostic microbiology has entered a new era [2]. The new technique offers revolutionary changes in the routine practice, allowing a faster and more accurate diagnosis of clinical isolates and reducing the need for experienced technologists [46]. Therefore, many microbiology laboratories in the developed world have implemented this new technology [47]. Still, changing to this technique might also carry some disadvantages, which need to be considered, especially in resource-limited regions such as South America [48]. Main drawbacks are the high costs of the equipment, the dependence on highly trained technical support and specific reactants, and the loss of knowledge and experience regarding traditional identification procedures [48]. Other important questions include the diagnostic performance with local spectrum of microorganisms and finally, the differences between the two available commercial systems [48]. Direct comparative studies of both systems are scarce and mostly derive from industrialized countries in Europe, Asia, and North America [16,37,49–52]. Therefore, there is a need for evaluations in non-industrialized regions using local microbial samples and routine laboratory infrastructure.

The two MALDI-TOF devices were temporarily implemented in our routine workflow during several months and were tested strictly in parallel. Both platforms identified the vast majority of tested isolates. However, Vitek MS showed a higher performance in the identification to species and species complex level, which is in accordance with previous studies [50,51]. This difference mainly based on better identification rates within Gram-positive cocci and yeasts (Tables 1 and 2), e.g. various strains of *Streptoccocus mitis* group were only identified to the genus level by Microflex LT, a limitation that was reported in earlier studies [46,53]. Microflex LT was also less accurate determining the species of coagulase negative staphylococci. However, in almost all of these cases, species results were correctly reported, but with a low identification score of 1.80 to 1.98 (S1 Table). This finding is in accordance with previous reports, some of which suggest lowering the cut-off to 1.7 to reduce the need for supplementary testing [51]. Other frequent Gram-positive cocci such as enterococci, *S*. *aureus*, and beta-hemolytic streptococci were correctly identified by both devices, as previously reported [51,54].

Another difference was observed in the species identification of yeast isolates. While Vitek MS correctly identified 98% (46/47) to the species level, Microflex LT achieved this in only 79% (37/47). Problems mainly occurred with *C*. *albicans* strains, which were categorized as *Candida* spp. due to low ID scores between 1,75 and 1,96 (Table 3, S1 Table). Similar to coagulase negative staphylococci, a lower validation score cut-off might improve accuracy without increasing misidentifications, as suggested by other studies [55]. In addition, Microflex LT required a specific extraction step for yeast isolates, which was laborious and operator dependent, probably explaining the lower identification rate of our study compared to others [46,51]. Nevertheless, both systems offer important advantages over traditional identification methods, permitting easier, cheaper, and timelier yeast diagnosis [55].

The majority of *Enterobacteriaceae* and other Gram-negative aerobic bacteria were correctly identified to the species or species complex level by both instruments. Some of the limitations within these groups were shared by both systems, e.g. the inability to differentiate very closely related species of the *E*. *cloacae*, *A*. *baumannii*, and *B*. *cepacia/cenocepacia* complexes. This was also observed with *B*. *pertussis* and *B*. *bronchioseptica* and among *Aeromonas* species. It is worth noting that 16S rRNA sequencing was also inconclusive in many of these difficult-to-identify species groups, as previously described [12,56]. Further investigations are needed to determine which of these limitations are inherent to the technique and which can be overcome with broader spectra databases.

Significant limitations affected the identification of certain enteropathogens including *Salmonella enterica*. The necessary serotyping within this species maintains a domain of conventional methods that cannot be reliably replaced by MALDI-TOF MS [57]. Still, the database of Vitek MS included certain *Salmonella* serotypes, and in our small panel, three of five typhoidal serotypes were correctly recognized (S1 Table), as in a previous report [37]. However, according to the manufacturer´s instructions, these identifications should be confirmed by conventional serotyping. Other problems affected the identification of *Shigella*, a main cause of pediatric diarrhea in Latin American countries [58]. At present, both systems are unable to distinguish between *Shigella* and *E*. *coli*, which, in the future, might be overcome by more advances databases [59]. Other enteropathogens such as *Yersinia*, *Campylobacter*, *Vibrio*, *Aeromonas*, and *Plesiomonas* were correctly identified by both systems with the above mentioned limitations of species differentiation among *Aeromomas* and the database-dependent limitation of Microflex LT to differentiate *V*. *cholera*.

A main advantage of MALDI-TOF technology for routine diagnosis is the accurate identification of certain microorganisms that, by classical methods, are mostly classified to the genus or even genus group level, e.g. coryneform bacteria, bacteroides group anaerobes, or fastidious Gram-negative rods [7,45,60]. With MALDI-TOF, these bacteria are now identifiable without the high costs and significant time span related to multiple biochemical tests and/or 16S rRNA analysis [60]. Our evaluation confirmed that both MALDI-TOF platforms rapidly and precisely identified most of such microorganisms. Clinically relevant Gram-positive rods such as *L*. *monocytogenes*, *E*. *rhusiopathiae*, *T*. *bernardiae* or *Corynebacterium* spp. were reliably identified. The identification of branched Gram-positive rods such as *Nocardia* spp. and *Actinomyces* spp. is a known challenge for conventional methods and even 16S rRNA sequencing [61]. The correct genus diagnosis of these bacteria in routine microbiology has improved with the application of MALDI-TOF [11]; still, species identification is not always reached due to slow amount of material, weak protein signals, and insufficient representation in the databases [8,22,62,63]. MALDI-TOF systems also offer new perspectives for the routine identification of anaerobes, avoiding the technical difficulties and time loss of traditional diagnosis [61]. In our study, Gram-positive and -negative anaerobes were identified with high accuracy, as previously observed [46]. The few strains (3/32) that reached only genus level by Microflex LT were due to identification scores slightly below the threshold, raising the question of possible cut-off modification as mentioned above (with coagulase-negative staphylococci and *Candida* spp.). The higher level of correct anaerobe identification compared to previous studies might reflect the updated databases used in our work [4,37].

It is important to take into consideration that the study was performed in 2012 and 2013 and that the described identification differences depended on the databases and software versions at that time, which have been updated in the meantime, e.g. Vitek MS was changed from Myla™ v2.0 to v3.0 in February 2017 in Chile.

As in other studies, the overall handling of both devices was judged as simple and required only few days of training [46,52]. In contrast to a recent study, our personnel preferred the smaller size and loading position of Microflex LT as well as the ease to prepare the smears [51]. The Microflex LT was also less noisy and gave faster individual results. For Vitek MS, the disposable targets and ready-to-use matrix solution, although associated with higher costs, were assessed as important advantages, since they required less hands-on time and experience. In accordance with other reports, the overall practical handling of Vitek MS was slightly favored by our technologists [51]. As a known advantage of MALDI-TOF technique, the operational costs of both platforms were much cheaper in comparison to traditional identification methods such as API or Vitek 2.

The overall workflow integration was less challenging with Vitek MS, since its software (Myla™) connected to the existing laboratory information system (K-Mic^®^, Kern, Buenos Aires, Argentina) without the need to develop a novel interface. Therefore, the integration to existing instruments (Vitek 2 Compact and XL bioMérieux) was rapid and simple. To our experience, high level information technology support and maintenance are a critical factor in Chile and, at the time of the study, local technical assistance was only available for Vitek MS. Remote assistance, which was offered by both providers, suffered several drawbacks such as language problems, time differences, and slow internet connectivity. Besides, technologists felt more confident with on-site help instead of solving a problem by phone or email.

Apart from the analytical performance, the above-mentioned issues such as connectivity, maintenance, and technical support are important determinants for the decision to implement a MALDI-TOF device [37]. Although these factors depend on the individual situation of each laboratory, they might pose much bigger challenges, and are often less discussed in studies from Europe and North America. In our case, for example, technical expertise on the highest level was only available abroad for both devices and not provided in Spanish language. Mutual back-up agreements with other laboratories using the same technology help to overcome technical problems or maintenance shortfalls. Other important conditions affecting laboratory automation in less developed settings include electricity and temperature variations, humidity, and availability of supplies and spare parts; the robustness and stability of MALDI-TOF technology under these circumstances should be reevaluated after longer periods of routine use.

## Conclusions

Both MALDI-TOF systems exhibited a high performance and robustness, being more rapid, accurate and cheaper diagnostic platforms than biochemical or molecular tests. The vast majority of local isolates was correctly identified by both devices, although Vitek MS reached a higher diagnostic accuracy for species and species complex identification, which mainly affected coagulase negative staphylococci and *Candida* species. Most likely, these differences reflected the stricter diagnostic criteria of the Microflex LT system.

The evaluation of user friendliness and other technical aspects was overall positive and showed only marginal differences. While Microflex LT exhibited some advantages in its practical handling, the global assessment slightly favored Vitek MS mainly due to its ready-to-use supplies, easier connectivity and integration to the existing workflow, and availability of local technical support.

Overall, MALDI-TOF MS represents a change of paradigm for clinical microbiology and has the potential to close the gap between technical facilities in industrialized versus non-industrialized countries.

## Supporting information

S1 Table Data of microbial identification using Microflex LT and Vitek MS plus molecular biology. (XLSX)
